# Satisfaction With Health Care by Dual Sensory Impairment Status

**DOI:** 10.1093/geroni/igaa057.2892

**Published:** 2020-12-16

**Authors:** Lama Assi, Ahmed Shakarchi, Bonnielin Swenor, Nicholas Reed

**Affiliations:** 1 Johns Hopkins Wilmer Eye Institute, Baltimore, Maryland, United States; 2 Johns Hopkins University, Baltimore, Maryland, United States

## Abstract

Sensory impairment is a barrier to patient-provider communication and access to care, which may impact satisfaction with care. Satisfaction with the quality of care received in the past year was assessed in the 2017 Medicare Current Beneficiary Survey (weighted sample=53,905,182 Medicare beneficiaries). Self-reported sensory impairment was categorized as no sensory impairment, hearing impairment (HI)-only, vision impairment (VI)-only, and dual sensory impairment (DSI) – concurrent HI and VI. In a model adjusted for sociodemographic characteristics and health determinants, having DSI was associated with higher odds of dissatisfaction with the quality of care received (Odds Ratio [OR]=1.53, 95%Confidence Interval [CI]=1.14-2.06) relative to no sensory impairment; however, having HI-only or VI-only were not (OR=1.33, 95%CI=1.94-1.89, and OR=1.32, 95%CI=0.95-1.93, respectively). These findings have implications for healthcare providers as Medicare shifts to value-based reimbursement. Moreover, previous work that singularly focused on HI or VI alone may have failed to recognize the compounded effect of DSI.

